# Streptozotocin-Induced Adaptive Modification of Mitochondrial Supercomplexes in Liver of Wistar Rats and the Protective Effect of *Moringa oleifera* Lam

**DOI:** 10.1155/2018/5681081

**Published:** 2018-03-01

**Authors:** María Alejandra Sánchez-Muñoz, Mónica Andrea Valdez-Solana, Mara Ibeth Campos-Almazán, Óscar Flores-Herrera, Mercedes Esparza-Perusquía, Sofia Olvera-Sánchez, Guadalupe García-Arenas, Claudia Avitia-Domínguez, Alfredo Téllez-Valencia, Erick Sierra-Campos

**Affiliations:** ^1^Facultad de Ciencias Químicas, Universidad Juárez del Estado de Durango Campus, Gómez Palacio, DGO, Mexico; ^2^Facultad de Medicina y Nutrición, Universidad Juárez del Estado de Durango Campus, Durango, DGO, Mexico; ^3^Departamento de Bioquímica, Facultad de Medicina, Universidad Nacional Autónoma de México, Mexico City, Mexico; ^4^Facultad de Ciencias de la Salud, Universidad Juárez del Estado de Durango Campus, Gómez Palacio, DGO, Mexico

## Abstract

The increasing prevalence of diabetes continues to be a major health issue worldwide. Alteration of mitochondrial electron transport chain is a recognized hallmark of the diabetic-associated decline in liver bioenergetics; however, the molecular events involved are only poorly understood. *Moringa oleifera* is used for the treatment of diabetes. However, its role on mitochondrial functionality is not yet established. This study was aimed to evaluate the effect of *M. oleifera* extract on supercomplex formation, ATPase activity, ROS production, GSH levels, lipid peroxidation, and protein carbonylation. The levels of lipid peroxidation and protein carbonylation were increased in diabetic group. However, the levels were decreased in *Moringa*-treated diabetic rats. Analysis of in-gel activity showed an increase in all complex activities in the diabetic group, but spectrophotometric determinations of complex II and IV activities were unaffected in this treatment. However, we found an oxygen consumption abolition through complex I-III-IV pathway in the diabetic group treated with *Moringa*. While respiration with succinate feeding into complex II-III-IV was increased in the diabetic group. These findings suggest that hyperglycemia modifies oxygen consumption, supercomplexes formation, and increases ROS levels in mitochondria from the liver of STZ-diabetic rats, whereas *M. oleifera* may have a protective role against some alterations.

## 1. Introduction

Mitochondria, which are mainly composed by proteins and lipids, are considered the most complex and the most important organelles of eukaryotic cells. They not only play a leading role in the energy metabolism, but also closely involve in many cellular processes [1]. Moreover, mitochondria are highly dynamic organelles that continuously divide and fuse as well as move within the cell [2]. In addition, it is now well established that the individual respiratory complexes can be organized into supercomplexes, but the composition and abundance of these may vary among organisms and tissues depending on the metabolic and physiological conditions [3–5] as well as on the lipid content of the mitochondrial inner membrane [6, 7]. However, mitochondria are a source of reactive oxygen species (ROS) which are involved in many pathological scenarios [8] and often play an essential role in physiological cell death mechanisms [9].

Mitochondrial dysfunction has recently been identified as a common metabolic defect associated with diabetes, obesity, and its metabolic complications [10, 11]. Previous studies have demonstrated that chronic diabetes induced by streptozotocin (STZ) provoked significant alterations in hepatic mitochondrial function which were restored to normality with insulin treatment [12] or with mifepristone (RU 38486) treatment [13]. In addition, it has been postulated that STZ-induced cytotoxicity in HepG2 cells is mediated, at least in part, by the increase in ROS and reactive nitrogen species (RNS) production, oxidative stress, and mitochondrial dysfunction [14]. Moreover, diverse studies suggest that mitochondrial oxidative function was compromised in diabetic and prediabetic humans as evidenced by reduced levels of fatty acid oxidation, insulin-stimulated ATP synthesis, and expression of genes involved in oxidative phosphorylation (OXPHOS) [15–17]. With respect to OXPHOS, activity was suggested that mitochondrial diabetes may also affect the complex V [18], and it is interesting to mention that, in diabetic patients' muscle, blue native gel electrophoresis revealed a striking decrease in complex I, III, and IV containing supercomplexes [19]. In addition, impairment of pyruvate dehydrogenase complex on the citric acid cycle and glucokinase activity during diabetes has been reported [19, 20]. These findings can be associated with an increased in ROS production and a decrease in cellular reduced glutathione (GSH) content in STZ-induced diabetic rats [21] and diabetic patients [22].

*Moringa oleifera* is commonly used in folk medicine as an antidiabetic agent via its antioxidant property. Yet, its biological activity is not limited to the antioxidant capacity. In fact, other important biological activities such as hypolipidaemic, antiatherosclerotic, and anticarcinogenic activities of *M. oleifera* leaves and seeds have been reported [23–26]. However, phenolic compounds found in *M. oleifera*, especially flavonoids, possess both antioxidant and prooxidant properties depending on concentration used. The latter which is exhibited at higher concentrations of phenolic compounds such as quercetin, galangin, taxifolin, catechin, and prenylated flavonoid have been shown to affect mitochondrial energetic processes (see supplementary materials available here) [27, 28]. In addition, it has been shown that mitochondria are a plausible main target of flavonoids mediating preventive actions against stress and mitochondrial dysfunction-associated pathologies [29]. Recent evidence indicates that *M. oleifera* aqueous leaf extract presents anticancerous effect on A549 cancer cells by affecting mitochondrial membrane potential and ATP levels [30]. More recently, Khan et al. [31] showed that aqueous extract of *M. oleifera* leaf protects pancreas against ROS-mediated damage by enhancing cellular antioxidant defenses and minimizing hyperglycemia in STZ-induced diabetes, which might be due to the glucose uptake enhancement in skeletal muscle, insulin secretion stimulation, and alpha-amylase and alpha-glucosidase inhibition. Thus, the favorable roles of *M. oleifera* in glucose metabolism and antioxidant system led us to investigate the effects of *M. oleifera* on diabetes-induced mitochondrial changes in liver. The aim of this study was to investigate the protecting effect of *M. oleifera* extract upon STZ-induced mitochondrial dysfunction. To assess the degree of injury of the STZ, both respiratory and enzyme activity parameters were evaluated and compared with the changes in the *M. oleifera*-treated group.

## 2. Materials and Methods

### 2.1. Preparation of the Extract

The extract was prepared using 23 g of dry-ground sample and 260 mL of 80% methanolic aqueous solution by successive maceration. The mixture was shaken in a magnetic grid at room temperature for 24 h and then filtered through Whatman filter paper number 1. The final extract was concentrated on a rotary evaporator, placed in a deep freezer for 24 h and lyophilized to obtain a powdered extract that was kept at −80°C.

### 2.2. Ethics Statement

All experiments were performed in compliance with the guideline for the welfare of experimental animals by the National Institutes of Health and in accordance with the guidelines of Institutional Animal Care. This study was approved by the Institutional Animal Ethics Committee at the Faculty of Health Science, UJED.

### 2.3. Diabetic Model and Treatment

Streptozotocin (STZ) was dissolved in a citrate buffer (0.1 M, pH 4.5) and intraperitoneally injected (55 mg/kg) to induce diabetes in rats. Rats injected only with citrate buffer served as control. Type 1 diabetes was confirmed evaluating fasting plasma glucose levels after 5 days of induction; the inclusion criteria to establish diabetes were 200 mg/dL of fasting plasma glucose. Rats were divided in control (C group), diabetic (D group), and *M. oleifera*-treated diabetic (M group) groups. M group was daily administered with a 200 mg/kg dose of extract by gavage during 3 weeks, and remaining groups were administered with water as vehicle.

### 2.4. Isolation and Purification of Mitochondria

The rat liver was collected immediately after euthanasia and homogenized in 100 mL of a buffer containing 20 mM Tris-HCl, 200 mM mannitol, 50 mM sucrose, 1 mM EDTA, 1 mM PMSF, 1 protease inhibitor tablet, and 0.1% bovine serum albumin (BSA) (pH 7.4; buffer A). Cellular and nuclear fractions were removed in the pellet by centrifuging at 3,500 rpm for 10 min at 4°C. Mitochondria were obtained by centrifuging the supernatant for 10 min at 11,000 rpm. Then, mitochondria were washed and resuspended in buffer A without BSA and centrifuged at 11,000 rpm for 10 min. Mitochondria were loaded on a Percoll gradient 15, 23, and 40% in buffer A without BSA and centrifuged for 35 min at 25,000 rpm at 4°C [32].

### 2.5. Oxygen Consumption

Oxygen uptake was estimated polarographically using a Clark-type electrode in a 1.5 ml water-jacketed chamber at 37°C. The mixture contained 250 mM sucrose, 20 mM HEPES, 50 mM K_2_HPO_4_, 10 mM H_3_PO_4_, 10 mM MgCl_2_, and 1 mM EGTA, 0.1% BSA (pH 7.4) [33]. Oxygen consumption was stimulated by the addition of 0.1 mM NADH or 10 mM succinate (in the presence of 2 *µ*M rotenone). Otherwise, artificial substrates such as ascorbate/TMPD (10 mM and 100 *µ*M, respectively, in the presence of 2 *µ*M antimycin) were used for complex IV activity, and malonate and KCN were added to inhibit complex IV and complex II (10 mM and 5 mM, respectively).

### 2.6. NADH Dehydrogenase and Succinate Dehydrogenase Activities

Activities of complex I (NADH : DCPIP oxidoreductase) and complex II (succinate : DCPIP oxidoreductase) were determined spectrophotometrically at 600 nm by following the reduction of the artificial electron acceptor 2,6-dichlorophenol-indophenol (DCPIP; 50 *μ*M; *ε*_DCPIP_ = 21 mM^−1^·cm^−1^). Mitochondria were permeabilized with 0.03% zwittergent and incubated in 10 mM KH_2_PO_4_, 5 mM MgCl_2_, 1 mM EGTA, and 120 mM KCl (pH 7.4), either with 0.2 mM NADH (complex I) or 2 mM succinate (complex II), plus 0.2 mM methosulfate phenazine (PMS). Mitochondria protein concentration was 1 mg/ml, and the reaction was started by the addition of NADH or succinate [34].

### 2.7. ATP Synthase Assay

ATP hydrolysis of complex V was measured spectrophotometrically at 25°C using a coupled assay to the oxidation of NADH (*ε*_340 nm_ = 6.22 mM^−1^·cm^−1^). The assay contained 100 *μ*g mitochondrial protein, 10 mM HEPES (pH 8.0), 100 mM NaN_3_, 100 *µ*M NO_4_Na, 90 mM KCl, 3 mM MgSO_4_; the ATP regenerating system consisted of 5 mM phosphoenolpyruvate, 2 mM ATP, 0.03% zwittergent, 50 units/mL pyruvate kinase, and 30 units/mL lactate dehydrogenase. The ATPase reaction was started by the addition of 0.1 mM NADH. Oligomycin (6 *µ*g/mL) was added to inhibit ATPase activity and verify F1F0-ATP synthase integrity; mitochondria were incubated with oligomycin for 30 min [35].

### 2.8. Native Electrophoresis

Respiratory complexes and supercomplexes were resolved by native PAGE as reported previously [36]. Purified liver mitochondria (1 mg) were suspended in 50 mM Bis-Tris and 500 mM 6-aminocaproic acid (pH 7.0) and solubilized by adding digitonin (detergent : protein ratio of 1 : 5). The mixtures were incubated for 30 min at 4°C and centrifuged at 100,000 g for 30 min. The supernatants were recovered and immediately loaded on a linear gradient polyacrylamide gradient gels (4–10%) for Blue Native PAGE (BN-PAGE) or Clear Native PAGE high resolution (hrCN-PAGE).

For BN-PAGE, the anode buffer contained 50 mM Bis-Tris/HCl (pH 7.0); the cathode buffer contained 50 mM tricine and 15 mM Bis-Tris (pH 7.0), and Coomassie (0.02%). For the hrCN-PAGE, the anode buffer contained 25 mM imidazole/HCl (pH 7.0); while the cathode buffer contained 50 mM tricine, 7.5 mM imidazole, 0.01% *β* dodecyl D-maltoside, and 0.05% sodium deoxycholate (pH 7.0), supplemented with Ponceau S red [37]. Gels were run at 4°C and 35 V for 16 h. The molecular weights of the respiratory complexes or supercomplexes were estimated by using digitonin bovine heart mitochondrial complexes as standard: single complex: I = 1,000 kDa, V = 750 kDa, III_2_ = 500 kDa, IV = 230 kDa, II = 130  kDa; supercomplexes: I-III-IV_1–4_ = 1500–2100 kDa, V_2_ = 1500 kDa.

### 2.9. Complex and Supercomplexes In-Gel Activities

The in-gel activity assays were performed as Wittig and Schägger [38] for complex I activity (NADH : methylthiazolyldiphenyl tetrazolium bromide reductase), complex II activity (succinate : methylthiazolyldiphenyl tetrazolium bromide reductase), and complex IV activity (cytochrome *c* : diaminobenzidine reductase). In all cases, the assays were performed at 20–25°C and stopped with 50% methanol and 10% acetic acid, after 10–25 min.

The in-gel activity of complex V was performed in 50 mM glycine (adjusted to pH 8.0 with triethanolamine), 10 mM MgCl_2_, 0.15% Pb(ClO_4_)_2_, and 5 mM ATP. ATP hydrolysis was correlated with the development of white lead phosphate precipitates. The reaction was stopped using 50% methanol, and subsequently, the gel was transferred to water and scanned against a dark background as described previously [39].

### 2.10. SDS-Gel Electrophoresis and Western Blot Analysis

Liver mitochondrial proteins (20 *μ*g per well) were separated by SDS-PAGE according to Laemmli [40] in a 10% polyacrylamide gel under denaturing conditions. Proteins were then transferred from gel to PVDF membrane (Immobilon P; Millipore, Bedford, MA) in a semidry electroblotting system (Bio-Rad) at 25 V for 50 min. Membranes were blocked in 500 mM NaCl, 0.05% Tween-20, and 20 mM Tris-base (pH 7.5) (TTBS buffer), containing 5% blotting grade blocker nonfat dry milk. Then, membranes were incubated with antitotal OXPHOS antibody cocktail (at 1/500 dilution). Immunoreactive bands were visualized by enhanced chemiluminescence (Amersham Life Science, Inc.), according to the manufacturer's instructions, using horseradish peroxidase-conjugated antimouse IgG (at 1/10,000 dilution), and densitometric analyses were performed with the software Image Studio Lite version 5.2 (LI-COR Biosciences).

### 2.11. Protein Determination

The protein levels were estimated by the method described by Lowry et al. using BSA as standard [41].

### 2.12. Mitochondrial Glutathione Reductase Activity

Glutathione reductase enzymatic activity was recorded by NADPH consumption. Briefly, 50 *µ*g of purified mitochondria was placed in a phosphate buffer (50 mM, pH 7.0) containing 1 mM GSH and 0.1 M NADPH. NADPH reduction was measured at 340 nm (*ε*_NADPH_ = 6.22 M^−1^·cm^−1^).

### 2.13. Measurement of Glutathione Concentration by HPLC-UV

To quantify GSH and GSSG concentrations, a standard curve of oxidized and reduced glutathione was used as described by Yilmaz et al. [42]. Mitochondrial samples were centrifuged at 500 rpm for 10 min and filtered to be injected onto a Kromasil ETERNITY C18 column (4.6 × 150 mm). Mobile phase containing 10 mM of monobasic sodium phosphate and 2% methanol (pH 3.0) was used at a flow rate of 1 mL/min in isocratic run. GSH and GSSG eluted from the column were detected at 210 nm.

### 2.14. Lipid Peroxidation Assay

Mitochondrial lipid peroxidation was estimated by the thiobarbituric acid reactive substances (TBARS) method consisting of TBA-TCA-HCl reaction as described by Buege and Aust [43]. Samples were boiled at 95°C for 60 min, followed by a cooling and centrifugation steps at 12,000 rpm for 10 min at 4°C. The pink product absorbance (formed when the MDA reacts with TBA) was spectrophotometrically recorded at 532 nm (*ε*_MDA_ = 1.56 × 10^5^ M^−1^·cm^−1^). MDA-TBA adduct peak was calibrated with tert-butyl hydroperoxide simultaneously processed as samples.

### 2.15. Mitochondrial H_2_O_2_ Measurement

H_2_O_2_ emission was determined by the fluorogenic indicator Amplex Red (Invitrogen) oxidation in presence of horseradish peroxidase as described by Starkov [44]. Fluorescence was recorded in a spectrofluorometer (LS 55 PerkinElmer Life Sciences) with excitation and emission wavelengths of 555 and 581, respectively. Briefly, 300 *µ*g of purified mitochondria was added to 1 mL incubation buffer containing 125 mM KCl, 20 mM Hepes, 0.2 mM EGTA, 2 mM KH_2_PO_4_, 2% BSA, 1 *µ*M Amplex Red, and 4 U horseradish peroxidase (pH 7.2). H_2_O_2_ production was initiated after addition of 5 mM pyruvate, 2.5 mM malate, and 10 mM succinate as substrates and 1 *µ*M rotenone, 0.2 *µ*M antimycin A, and 5 mM malonate as inhibitors.

### 2.16. Measurement of Protein Carbonylation

Determination of carbonyl content was followed as Levine et al. [45]. The oxidative damage to proteins was determined by carbonyl groups based on their reaction with 2,4-dinitrophenylhydrazine (DNPH) to form hydrazones. Briefly, 0.5 mg of mitochondria was incubated with 20 mM DNPH solution for 1 h; then proteins were precipitated with 20% (w/v) of trichloroacetic acid and redissolved in DNPH. In brief, the proteins were precipitated by the addition of 20% (w/v) of trichloroacetate; protein pellet was washed three times with ethanol : ethyl acetate (1 : 1) and resuspended in 1 mL of 6 M guanidine. The absorbance was recorded at 370 nm (*ε*_Hydrazone_ = 22 × 10^3^ M^−1^·cm^−1^).

### 2.17. Measurement of HO-1 Activity

Fresh livers were placed in prechilled Dounce homogenizer, and cold homogenization buffer containing 100 mM potassium phosphate buffer (pH 7.4), 2 mM MgCl_2_, 250 mM sucrose, and a protease inhibitor cocktail (10 *μ*g/mL leupeptin, 10 *μ*g/mL trypsin inhibitor, 2 *μ*g/mL aprotinin, and 1 mM PMSF) was added. The homogenate was centrifuged at 10,000 g for 30 min at 4°C, followed by the supernatant centrifugation at 100,000 g for 60 min at 4°C, to obtain the microsomal fraction as a pellet. HO-1 activity was spectrophotometrically measured as described previously [46]. The microsomal fraction (50 *μ*L) was added to the reaction mixture (500 *μ*L) containing 0.8 mM NADPH, 2 mM glucose-6-phosphate, 0.2 unit of glucose-6-phosphate dehydrogenase, 20 *μ*M hemin, 100 mM potassium phosphate buffer (pH 7.4), and 2 mg of rat liver cytosol as a source of biliverdin reductase. The mixture was incubated at 37°C for 60 min in dark, and samples were left in an ice bath for at least 2 min to stop the reaction. Bilirubin product was determined by calculation from difference in optical density (OD) at 464 nm and 530 nm (OD_464_–OD_530_ nm) of the sample. HO activity is expressed as pmol/min/mg protein.

### 2.18. Data Analysis

The obtained data are represented as mean ± standard deviation of three independent determinations, using the Sigma Plot software version 11.0. Differences between means were obtained by analysis of variance (ANOVA) and multiple comparison tests. *P* values < 0.05 were considered as significant.

## 3. Results and Discussion

The effectiveness of *M. oleifera* extract in alleviating diabetes was assessed in the STZ-induced diabetic model in Wistar rats. In response to STZ, rats showed increased water uptake, increased urine production, increased blood glucose levels, and reduced weight gain (D group = 229 ± 9.05 mg/dL and 156 ± 12 g), which were unaltered in the control group (C group = 78 ± 5.5 mg/dL and 187 ± 18 g), while M group significantly alleviated all parameters of diabetes (86 ± 4.2 mg/dl and 194 ± 8 g). These results suggest that *M. oleifera* leaf may be a potential agent in the treatment of type 1 diabetes and are agreed with the observations that suggest the beneficial effects of *Moringa oleifera* supplementation on diabetes [47, 48]. Hence, these results led us to investigate the valuable effects of the leaf extract on STZ-induced mitochondrial changes, in liver, evaluating STZ injury on both, respiratory and enzyme activities from respiratory chain and some of the antioxidant system comparing them with those from *M. oleifera* treatment.

### 3.1. *M. oleifera* Attenuates Oxidant Stress and the Decrease in the Glutathione System in Liver Mitochondria

Diabetic cells and tissues have the capacity to invoke adaptive mechanisms that evolved to defend against oxidative stress [49]. One putative mechanism is a defense system that would protect against ROS into mitochondria. These include the superoxide conversion to hydrogen peroxide (H_2_O_2_) by manganese superoxide dismutase (SOD) and scavenging H_2_O_2_ by catalase, glutathione peroxidase (GPx), or peroxiredoxin III [50]. Reduced glutathione (GSH) scavenges H_2_O_2_ via GPx, ubiquitously expressed both in the mitochondria matrix and intermembrane space [51]. In turn, the reduction of oxidized glutathione (GSSG) to GSH is catalyzed by glutathione reductase (GR), which requires NADPH. Thus, increased ROS removal results in increased NADPH turnover. Also, GSH can also be used in conjugation reactions to protect mitochondria enzymes from various toxins, for example, by-products in lipid peroxidation such as 4-hydroxynonenal (HNE) [52].

To assess the influence of STZ injection on redox state, mitochondrial GSH levels and GR of liver were examined. A single dose of STZ caused a significant decrease in GSH and total GSH contents of diabetic rats (Table 1). Basal levels of total GSH were 178.8 ± 5.7 *μ*mol/mg of protein in control mitochondria, whereas total GSH levels in isolated mitochondria from STZ-treated rats (D group) were significantly decreased by 70% compared with control (100.2 ± 1.3 *μ*mol/mg of protein). In contrast, *M. oleifera* treatment prevented a STZ-mediated decrease in GSH levels (M group = 223.7 ± 2.9 *μ*mol/mg of protein), which correspond to an increase of 25% compared with C group. It is worth to mention that M group rats showed a significant increase in values of GSH, total GSH (2 times), and GSH/GSSG ratio (*P* < 0.05) compared with D group. However, the M group ratio was 12 times reduced with respect to C group (Table 1). One possible explanation for this phenomenon may be the inactivation of mitochondrial GR activity. However, as observed in Table 1, STZ administration did not alter the GR activity in liver mitochondria when compared with control rats. However, M group samples significantly increased GR activity when compared with values of D and C groups (Table 1). Hence, these results show that GR inactivation is not the main mechanism of GSSG accumulation into the mitochondria.

The oxidative stress implications in diabetes pathogenesis are suggested to be produced not only by ROS generation but also by a nonenzymatic protein glycation, autoxidation of glucose, impairment of antioxidant enzymes, and peroxides formation. Therefore, GSH level decline is associated with oxidative damage to macromolecules, such as lipids and proteins. ROS-mediated lipid peroxidation is a crucial factor in the development of diabetic liver complications. In addition, GSH depletion induces heme oxygenase-1 (HO-1), a key microsomal enzyme in heme degradation to carbon monoxide (CO), iron (Fe^2+^), and biliverdin; this latter being converted into bilirubin by the cytosolic biliverdin reductase [53, 54]. Moreover, some observations suggest the cytoprotective mechanism of HO-1 against oxidative stress involving an increase in mitochondrial carrier levels and antiapoptotic proteins as well as in cytochrome *c* oxidase activity [55].

In order to evaluate this possibility, we measure carbonyls concentration, lipoperoxidation, and HO-1 activity. As observed in Table 2, C group showed the lowest levels of carbonylation and MDA. In contrast, STZ treatment increased lipid peroxidation and protein carbonyl content (Table 2). Besides, M group showed a significant decrease in lipoperoxidation in liver mitochondria (*P* < 0.05) when compared with D group. The carbonylation level in mitochondria of M group was significantly lower and showed significant difference when compared with D group (Table 2). In contrast, *M. oleifera* extract administration did not prevent the HO-1 induction provoked by STZ, where its enzymatic activity remained significantly higher. Our results clearly demonstrated that *M. oleifera* significantly suppressed both lipoperoxidation and protein carbonylation. However, *M. oleifera* did not lower the HO-1 activity, and little is known about the molecular mechanisms responsible for its activation, which requires further investigation.

It has been reported that *M. oleifera* exhibits bifunctional antioxidant properties related to its ability to react directly with ROS and to induce antioxidant enzymes expression such as superoxide dismutase, catalase, glutathione reductase, and glutathione peroxidase [56–58]. We confirmed previous data and showed that *Moringa* not only decreased lipoperoxidation and protein carbonylation levels in rat livers but also increased HO-1 activity, parameters associated with a cytoprotective mechanism against oxidative stress [59].

### 3.2. Effects of STZ and *M. oleifera* on Oxygen Consumption

Although many previous studies have reported pharmacological properties of *M. oleifera*, particularly as antioxidant and antidiabetic properties that may provide benefits for diabetic patients [25, 60, 61], there are no reports that show *M. oleifera* extract effect on mitochondria functionality. To determine the changes of mitochondrial respiration in STZ-induced diabetic rats and *M. oleifera* protective effect, we measured the mitochondrial respiratory chain using Clark-type oxygen electrode and determined enzymatic activity of each complex by spectrophotometric methods.

**Figure 1:**
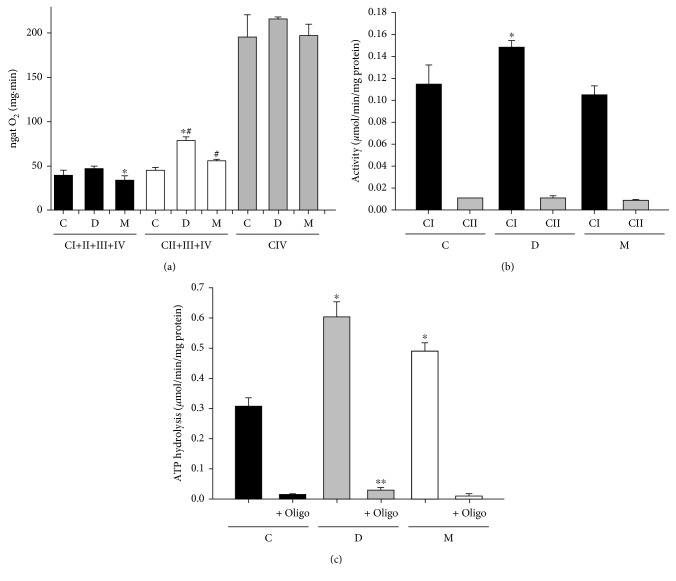
*Moringa oleifera* effect on mitochondrial respiratory chain. (a) Oxygen consumption (^∗^ *P* < 0.05 versus D; ^#^ *P* < 0.05 versus C); (b) enzymatic activity of complex I and II (^∗^ *P* < 0.05 versus C and M); (c) F_1_/F_0_ ATPase activity (^∗^ *P* < 0.05 versus C and M; ∗^∗^ *P* < 0.05 versus M + oligo) from liver mitochondria. C: control; D: diabetic; M: *Moringa*-treated diabetic groups.

Figure 1(a) shows the respiratory activity of all groups in presence of complex I, II, and IV substrates. The combination of pyruvate + malate indirectly investigates the monocarboxylate and dicarboxylate transporters and the pyruvate dehydrogenase activities. The substrate combination produces NADH which donates electrons to complex I. In D group, the state 4 respiration with pyruvate + malate was not affected. However, *Moringa* treatment resulted in 15% decrease in the state 4 respiration. By contrast, succinate donates electrons to FAD^+^ in complex II and yields significantly high respiration state 4 rates in both D and M groups compared with C group. Otherwise, in diabetic rats, state 4 respiration with succinate increased by 80% compared with control rats. The observed change in the diabetic animals was rectified by *Moringa* treatment (Figure 1(a)). Additionally, functional analysis of complex IV (cytochrome *c* oxidase) maximal activity was assayed with ascorbate (Asc) and N,N,N′,N′-tetramethyl-p-phenylenediamine (TMPD), which is an artificial redox mediator that assists the electron transfer from ascorbate to cytochrome *c*. Complex IV respiration was calculated as the portion sensitive to cyanide potassium (KCN), a specific inhibitor of cytochrome *c* oxidase (Figure 1(a)), revealing no differences among all experimental groups.

Additionally, we measured the specific activities of respiratory chain complexes in liver mitochondria. Spectrophotometric analysis showed a significant increase in complex I and ATPase activities in D group (Figure 1(c)), while *Moringa* treatment was effectively reversing this alteration nearly to control values. Complex II showed no significant change in mitochondrial fraction of D group (Figure 1(b)). These data show that individual activities of mitochondrial electron transport chain (ETC) enzymes were not negatively modified in diabetic treatment. In addition, the respiratory properties of D and M groups have approximately 1.5–2 times succinate-respiratory rates compared with that of C group. Therefore, our results suggest that respiratory complex activities were not decreased in liver mitochondria in STZ-induced diabetic rats. These findings, which may appear, at first glance, contradictory, may be interpreted in terms of higher ETC efficiency, thus, avoiding energy losses by electron leakage in response to change in physiological functions and body energy requirements; ETC undergoes some modifications either during pathology development or disease [62]. In addition, several studies concerning STZ-treated rats have been performed with animals of different strains and different amounts of STZ [63, 64]. In addition, in isolated hepatocytes, increasing glucose concentration does not increase Δ*µ*_H+_, mitochondrial respiratory rate, or cytosolic NADH/NAD^+^ ratio; instead, most of glucose excess is converted to glycogen [65]. In fact, some authors have recently suggested that mitochondria overstimulation is a probable risk factor for insulin resistance, while moderate mitochondrial dysfunction may actually be protective under certain conditions, suggesting the mitochondrial modulation as a prospective therapy for metabolic diseases [66]. For this reason, it is important that future research clarifies the true energy functional state of isolated mitochondria from diabetic animals [67].

### 3.3. Modulation of Mitochondrial Complexes by STZ

**Figure 2:**
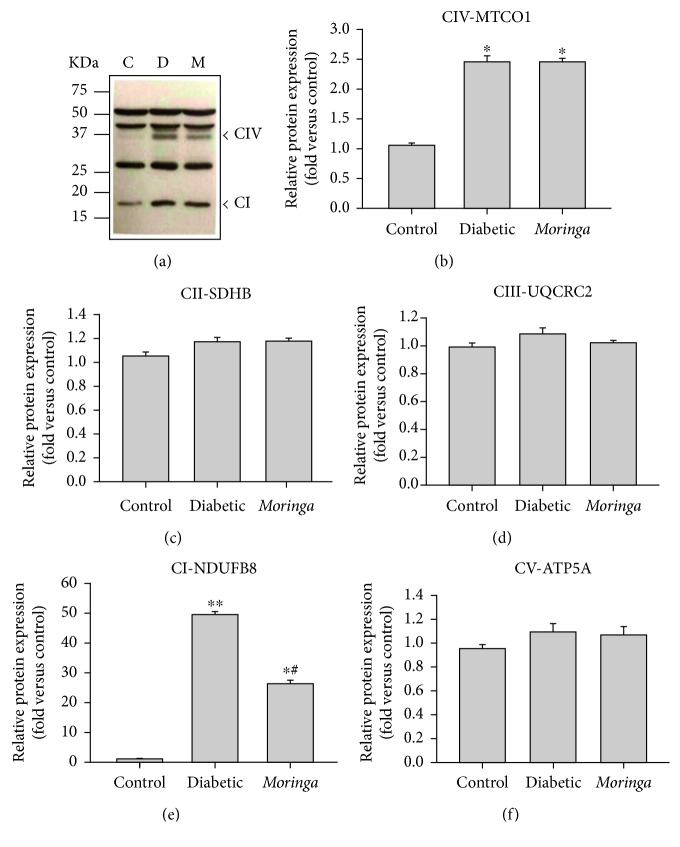
Characterization of OXPHOS proteins expressed in liver mitochondria during diabetes. (a) OXPHOS cocktail specificity demonstrated by a Western blot from liver-isolated mitochondria of diabetic rats and treated with *Moringa* extract. Relative expression of (b) MTC01 subunit of CIV, (c) SDHB subunit of CII, (d) UQCRC2 subunit of CIII, (e) NDUFB8 subunit of CI, and (f) ATP5A subunit of CV was performed by densitometric analysis from gel. Molecular mass standards are shown on the left panel of the gel. C: control; D: diabetic; M: *Moringa*-treated group. Data are shown as mean band density normalized relative to UQCR2. Significant differences are represented by ^∗^ *P* < 0.05 versus C; ^#^ *P* < 0.05 versus D.

As mitochondrial content can substantially impact on respiratory capacity, protein components of individual respiratory complexes were quantified. To test whether hyperglycemia and *M. oleifera* extract altered the composition of the ETC, we analyzed the expression level of nuclear-encoded mitochondrial complex I subunit NDUFB8, complex II subunit SDHB, complex III UQCRC2, complex IV MTCO1, and complex V ATP5A of each group. Interestingly, in diabetic rats, the NDUFB8 subunit resulted in an increase of complex I expression, while complex II and III were unaltered (Figure 2). These data which resulted in increased expression of NDUFB8 and MTCO1 support the suggestion that increased activity of mitochondrial respiratory chain could result from a proteome alteration leading to modulation of expression/activity of a range of mitochondrial components. More importantly, an upregulation of hepatic CI-NDUFB8 and CIV-MTCO1 was found in diabetic rats, consistent with changes in-gel activity of these complexes. Thus, it is plausible that increased NDUFB8 and MTCO1 contents observed in the STZ group, resulting from diabetes mellitus type 1, may account, in part, for the mitochondrial morphological changes observed, which could have downstream effects on mitochondrial functionality leading to hepatic dysfunction.

### 3.4. Loss of Redox State Does Not Destabilize Mitochondrial Supercomplexes

It is now widely accepted that mitochondrial respiratory chain is organized with stable and functional entities called supercomplexes (SC) [68]. SC consist of various ratios of copies of individual complexes (I, III, IV, and V) to form stable, supramolecular structures; for instance, CI forms a supercomplex with CIII_2_ and CIV (known as the respirasome), as well as with CIII_2_ alone (SC I + III_2_). CIII_2_ forms a supercomplex with CIV (SC III_2_ + IV), and CV forms dimers (CV_2_). In addition, another recent advance is that the discovery of respiratory megacomplex (MC I_2_III_2_IV_2_) represents the highest-order assembly of respiratory complexes [69], and it allows mitochondria to respond to energy requiring conditions and to minimize ROS generation during electron transfer reactions [70], as well as the sequestering of vulnerable sites of mitochondrial complexes from oxidative damage as a protective mechanism that prevents tight interactions between the individual complexes [71].

It is fairly well established in rectus abdominis muscle of diabetic obese patients. BN-PAGE revealed a striking decrease in complex I, III, and IV containing mitochondrial SC [72]. According to these results, Lenaz and Genova [71] suggest that oxidative stress acts primarily by disassembling supercomplex associations thereby establishing a vicious circle of oxidative stress and energy failure, ultimately leading to cell damage and disease.

It is interesting to mention that there are diverse specific regulatory proteins for the supramolecular organization of individual complexes that include CIV [73], respiratory SC factors 1 and 2 (Rcf1 and 2) [74], protein Cox interacting (Coi) [75], and COX7a2L [76]. These proteins downregulation can impair the formation of SC; for instance, some studies show that diverse pathologies decrease CIV subunit levels affecting stoichiometry and assembly of SC [77, 78]. In addition, diabetes induces mitochondrial genome damage by an increased free radical production depleting antioxidant status [79]. Moreover, other structural components as cardiolipin have been shown to be crucial for functionality and SC formation and might be involved in the pathophysiology of diabetes [80]. Thus, the impact of complex IV failure and other enzymes may cause an energy crisis due to a lower ATP synthesis and an increased ROS production.

**Figure 3:**
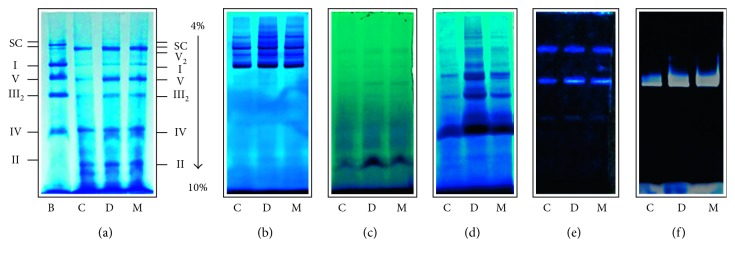
Electrophoretic representative pattern of liver mitochondrial solubilized of the different groups (5 g de digitonin/g protein). (a) Blue native polyacrylamide gel electrophoresis (BN-PAGE) stained with Coomassie blue; (b) complex I in-gel activity; (c) complex II in-gel activity; (d) complex IV in-gel activity; (e) complex IV in-gel activity; (f) high resolution clear native polyacrylamide gel electrophoresis (hrCN-PAGE) of complex V. B: bovine heart solubilized mitochondria (positive control); C: control; D: diabetic; M: *Moringa*-treated diabetic groups.

Figure 3(a) shows the Coomassie blue staining of the gels for all treatments and the colorimetric enzymatic staining of NADH, Succinate, COX, and ATPase complexes after detergent extraction and BN-PAGE or hrCN-PAGE (only for CV).  Figure 3(b) clearly indicates that the major form of supercomplexes is present in all samples. In contrast, the amount of free complex I and IV were decreased in D group, and these values did not change in mitochondria isolated from M group. Otherwise, the in-gel activity of complex II was significantly lower in C group compared with those in the D and M groups. In addition, the brown bands indicate the presence of complex IV in all groups and its increase in D group (Figure 3(d)). On the other hand, in-gel ATP hydrolysis/lead phosphate precipitation assay revealed bands representing the F_1_F_0_ monomer and F_1_F_0_ dimer bands in Figures 3(e) and 3(f) showing the same functional patterns as the in-solution assays, indicating the level of intrinsic activity driven by complex V. In support, we have shown by comparing the in‐gel enzyme activities that the ATPase activity of the F_1_F_0_‐ATP synthase is specifically and significantly increased in D group when compared with C group.

Our results are concerned with the changes in the amount of CIV subunits; for example, Cox6b1 is involved in the regulation of mitochondrial function by promoting SC formation, suggesting its antiaging effects of calorie restriction [81]. In addition, heart failure in dogs induced by coronary microembolism resulted in loss of complex IV containing SC of the electron transport chain [77, 78]. Similarly, in RAW 264.7 macrophages, knockdown of either subunit cytochrome *c* oxidase (CcO) Vb or CcO IV resulted in a significant decrease in CcO containing supercomplexes [78]. Liver mitochondria from ethanol-treated rat also showed a lower level of supercomplexes with a concomitant loss of CcO protein [82]. Therefore, complex IV has been shown to be necessary for maintaining the stability of complex I in SC, as shown in mouse fibroblast cell lines, where a reduced expression of subunit IVi1 or nonsense mutation in subunit I not only resulted in lower CcO content but also caused significant reduction in complex I [83]. Structural defects in complex III also affected the amount of complex I, whereas chemical inhibition did not. Patients with defects in cytochrome *b* not only lose complex III but also show decreased amounts of complex I, while maintaining a normal enzymatic activity [84]. Conversely, the disruption of complex I function caused by nonsense mutations in NDUFS4, a subunit of this large multimeric complex, led to the partial loss of complex III activity in skin fibroblast cultures obtained from Leigh-like patients [85, 86]. However, defects in the complex I subunit ND5 did not cause a loss of complex III in the I-III supercomplex [87].

### 3.5. H_2_O_2_ Production by Liver Mitochondria Oxidizing Complex I and Complex II Substrates

Several studies have reported that ROS overproduction by mitochondrial ETC is responsible for hyperglycemia-induced oxidative stress and the pathogenesis of diabetic complications [88, 89]; however, it is not clear whether mitochondria of diabetic origin really generate ROS independently of the surrounding diabetic milieu. Herlein et al. [90] showed that the gastrocnemius, heart, and liver mitochondria of STZ-diabetic rats were not irrevocably altered to produce superoxide excess either by complex I or complex III. Moreover, gastrocnemius and heart mitochondria demonstrated an increased respiratory coupling, instead of a decrement. In addition, mitochondria of insulin-deficient diabetic rats did show signs of ROS overproduction. Thus, the detailed molecular mechanism and sites of ROS generation during diabetes are controversial.

**Figure 4:**
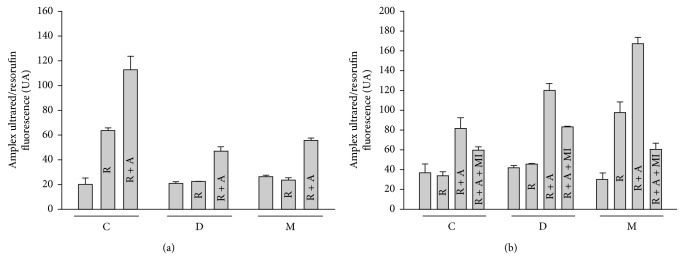
H_2_O_2_ production by mitochondrial respiratory chain measured by Amplex Red (UA) in liver mitochondria from different treatments (C: control; D: diabetic; M: *Moringa*-treated diabetic groups) oxidizing (a) pyruvate plus malate or (b) succinate as substrates and the effects of respiratory chain inhibitors (R: rotenone; A: antimycin A; Ml: malonate). Mitochondria were studied during state 4 respiration. To correct for the increase in background fluorescence of the Amplex Red/HRP detection system overtime, fluorescence was monitored for a period of ten minutes. This background was subtracted from resorufin trace. Data are means ± SEM (*n* = 5).

In isolated mitochondria, the rate of mitochondrial ROS generation is directly governed by membrane potential (Δ*Ψm*) and pH gradient across the inner membrane, favored only by a state 4 condition [91]. Hence, H_2_O_2_ production rate was measured in liver mitochondria using fluorescent dye Amplex Red and pyruvate plus malate or succinate, as complex I-III and II-III linked substrates, respectively, and the results are shown in Figure 4. Data presented in Figure 4(a) show that the major source of ROS is complex I for liver mitochondria of C group, using pyruvate plus malate as substrate, reflecting the generation of superoxide anion. In contrast, with M group-isolated mitochondria oxidizing succinate in state 4 produced 6 times more ROS compared with other treatments (C and D groups) (Figure 4(b)). Therefore, the mitochondrial ROS production rates varied dramatically among the three experimental groups in response to addition of respiratory inhibitors.

At the level of ROS formation, all groups have the same basal formation using malate plus pyruvate or succinate, but the addition of respiratory inhibitors had a varied effect on ROS production in the different groups. In case of the C group, the addition of rotenone and antimycin stimulated ROS production by using pyruvate and malate (Figure 4(a)). However, in both D group and M group, the addition of rotenone has no effect, while antimycin caused only a slight increase in ROS formation (Figure 4(a)). Nevertheless, with succinate, inhibitors have a different pattern. In the C group, the addition of rotenone causes no effect on ROS production, and antimycin favors its increase; but in this case, malonate has no effect (Figure 4(b)). However, in the diabetic group, antimycin has a greater effect on ROS production than control, and malonate adversely affects ROS formation (Figure 4(b)).

Finally, M group sensitivity to individual training inhibitors was unaltered in case of rotenone and malonate, but adding antimycin in this treatment favored ROS production (Figure 4(b)). Thus, measurements of ROS with Amplex Red cannot be used for sites of ROS generation from liver mitochondria treated with STZ and/or *M. oleifera*. This situation could be attributable to experimental conditions because complex I (rotenone), complex II (malonate), and complex III (antimycin A) inhibitors have been commonly used. However, the final concentration being used is not stationary, causing experimental errors that are different from one method to other. In addition, it is necessary to use other respiratory inhibitors, as stigmatellin and myxothiazol. No obstant, this does not deny other possible explanations that can affect ROS production as differences in the stoichiometry-activity ratios of the respiratory complexes [92], the susceptibility to proton pump slip at complex IV [93], or other mechanisms.

Damage to complex I, the most vulnerable ETC complex, increases ROS production, leading to a vicious circle of further mitochondrial dysfunction. It is important to note that complex I injury has a stronger impact on mitochondrial function compared with the damage to other complexes because mitochondria possess smaller amounts of complex I than other ETC complexes [94]. Superoxide production by complex I is much higher during reverse electron transport from succinate to NAD^+^ [95]. In addition, it was found that defective complex I produces more ROS [96], suggesting that structural modifications of the enzyme may play a crucial role in ROS production process. Recently, it was reported that pancreatic mitochondrial complex I showed aberrant hyperactivity in type 1 and 2 STZ-diabetic mice and rat and in cultured *β* cells [97]. Further experiments focusing on STZ-induced diabetes in rats revealed that complex I′s hyperactivity could be attenuated by metformin. Interestingly, in this study, no changes were reported in complex I activity in brain, liver, and heart by BN-PAGE [97]. However, the reason why complex I activity did not exhibit detectable increases in these tissues is unknown.

Our results in Figure 1(b) show that complex I activity was significantly higher in diabetes than in healthy individuals. This increased activity was apparently contributed by an increased NDUFB8 subunit protein content as shown in Figure 2(e). Moreover, the complex I hyperactivity also imposed pressure in complex IV (Figures 2(b) and 3(d)). These findings suggest that these elevated activities could be attributed for ROS production, given that higher ETC activity can also increase mitochondrial ROS generation [98, 99]. However, our results on specific sites of ROS generation along with ETC are controversial (Figure 4). This may explain why in some tissues seem that inhibition of electron transfer at complex I (by rotenone) may generate an increase in radical formation, whereas, in others, rotenone will reduce radical generation by preventing passage of electron further into the distal part of the chain. However, the basis for such a difference is obscure and presumed to be related to Δ*Ψm* changes and radicals leakage across the membranes [100].

Polyphenols have been traditionally viewed as antioxidants; however, increasing evidence has emerged supporting the ability of certain polyphenols to exert numerous ROS-scavenging independent actions. Then all these natural compounds modulate mitochondrial functions by inhibiting organelle enzymes or metabolic pathways, by altering the production of ROS and modulating the activity of transcription factors which regulate the expression of mitochondrial proteins [101]. Thus, some particular polyphenols are now recognized as molecules capable of modulate pathways that define mitochondrial biogenesis (i.e., inducing sirtuins), mitochondrial membrane potential (i.e., mitochondrial permeability transition pore opening and uncoupling effects), mitochondrial electron transport chain and ATP synthesis (i.e., modulating complex I to V activity), intramitochondrial oxidative status (i.e., inhibiting/inducing ROS formation/removal enzymes), and ultimately mitochondrial-triggered cell death (modulating intrinsic apoptosis) (review in [102]). Thus, some studies have indicated that mitochondria may be the target organelle of phenolic compounds [103, 104]. Recently, it was reported that galangin (natural flavonoid) could maintain liver mitochondrial function in diabetic rats through oxidative stress reduction and both antioxidant enzymes and respiratory complexes activities enhancement [79]. Therefore, the likely role of mitochondrial ROS in diabetes has led to efforts for developing effective antioxidant compounds targeted to mitochondria.

This study was designed to investigate the protective effect of *M. oleifera* on liver bioenergetics and to elucidate its potential mechanism. *M. oleifera* resulted in a well-preserved mitochondrial redox potential, significantly by elevating heme oxygenase-1 and decreasing ROS formation and lipoperoxidation. These observations indicated that STZ-induced mitochondrial oxidative damage was remarkably attenuated. Thus, to our knowledge, we have shown for the first time that *M. oleifera* extract modulates mitochondrial respiratory activity, an effect that may account for some of the protective properties of phytochemicals. These effects may be of physiological significance since it seems that some phytochemicals are concentrated into mitochondria. The results also support a pharmacological use of *M. oleifera* extract in drug to reduce mitochondrial damage in vivo. However, the details about mechanism of action require further investigation.

## 4. Conclusions

We provide experimental evidence indicating that *M. oleifera* extract targeting mitochondria can be used therapeutically to alleviate diabetes. Therefore, it will be important to identify regulatory proteins involved in the adjustment of respiratory chain complex organization/activity in response to altered redox state. In liver, the alteration of mitochondrial enzymatic activities and oxidative stress induced by STZ suggested of a compensatory response. In addition, *M. oleifera* extract upregulated mitochondrial genes linked with respiratory chain. Our data show an increased mitochondrial function and activity/expression of respiratory complexes in liver of STZ-diabetic rats, which can be normalized by *M. oleifera* at levels that do not markedly alter the consequences of hyperglycemia.

## Figures and Tables

**Table 1:** GSH and GSSG levels by HPLC-DAD and GR enzymatic activity in liver mitochondria from different groups.

Group	GSH (*µ*mol/mg protein)	GSSG (*µ*mol/mg protein)	GSH/GSSG ratio	Total GSH (*µ*mol/mg protein)	GR (U/min)
C	174.1 ± 35.1	4.8 ± 3.4	36.2 ± 0.19	178.8 ± 5.7	267.2 ± 11.7
D	50.4 ± 1^∗^	49.8 ± 1.1^∗^	1 ± 0.08^∗^	100.2 ± 1.3^∗^	236 ± 14.5
M	169 ± 1.2^∗∗^	54.7 ± 2.2^∗∗^	3 ± 0.05^∗∗^	223.7 ± 2.9^∗∗^	366.7 ± 23.8^∗∗^

C = control; D = diabetic; M = diabetic plus *Moringa*. ^∗^Significant difference versus control (*P* < 0.05). ^∗∗^Significant difference versus control and diabetic (*P* < 0.05).

**Table 2:** Levels of MDA and carbonyl groups in liver mitochondria from different treatments.

Group	MDA (nmol/mg prot)	Carbonyl groups (nmol/mg)	HO-1 (pmol/min/mg)
C	0.4317 ± 0.009	3.7232 ± 0.57	40.9 ± 4.9
D	0.5028 ± 0.06	12.738 ± 0.28^#^	85.7 ± 2.1^∗^
M	0.3851 ± 0.02^∗^	4.2645 ± 0.98	105.2 ± 3.4^∗^^#^

C = control; D = diabetic; M = diabetic plus *Moringa*. ^∗^Significant difference versus diabetic (*P* < 0.05). ^#^Significant difference versus control (*P* < 0.05).

## Supplementary Materials

Supplementary Materials Figure S1: Possible mitochondrial processes that are modulated by *Moringa oleifera* extract. It is now well established that the individual mitochondrial respiratory complexes can be organized into supercomplexes, but the composition and abundance of these may vary among organisms and tissues depending on the metabolic and physiological conditions. Alteration of mitochondrial electron transport chain is a recognized hallmark of the diabetic-associated decline in liver bioenergetics; however, the molecular events involved are only poorly understood. *Moringa oleifera* is used for the treatment of diabetes. However, its role on mitochondrial functionality is not yet established. This study was aimed to evaluate the effect of *M. oleifera* extract on supercomplex formation, ATPase activity, ROS production, GSH levels, lipid peroxidation, and protein carbonylation. The levels of lipid peroxidation and protein carbonylation were increased in the diabetic group. However, the levels were decreased in *M. oleifera*-treated diabetic rats. Analysis of in-gel activity showed an increase in all complexes activities in the diabetic group, but spectrophotometric determinations of complex II and IV activities were unaffected in this treatment. However, we found an oxygen consumption abolition through complex I-III-IV pathway in the diabetic group treated with *Moringa*. Respiration with succinate feeding into complex II-III-IV was increased in the diabetic group. We have shown for the first time that *M. oleifera* extract modulates mitochondrial respiratory activity, an effect that may account for some of the protective properties of phytochemicals. These effects may be of physiological significance since it seems that some phytochemicals are concentrated into mitochondria. The results also support a pharmacological use of *M. oleifera* extract in drug to reduce mitochondrial damage in vivo.
