# Supplementary material for: Streptozotocin-Induced Adaptive Modification of Mitochondrial Supercomplexes in Liver of Wistar Rats and the Protective Effect of Moringa oleifera Lam
Source: Biochem Res Int. 2018 Mar 1;2018:5681081. doi: 10.1155/2018/5681081 (PMC5852898; doi:10.1155/2018/5681081)

Graphical Abstract

S1 Fig. *Possible mitochondrial processes that are modulated by Moringa oleifera extract*

It is now well established that the individual mitochondrial respiratory complexes can be organized into supercomplexes, but the composition and abundance of these may vary among organisms and tissues depending on the metabolic and physiological conditions. Alteration of mitochondrial electron transport chain is a recognized hallmark of the diabetic-associated decline in liver bioenergetics; however, the molecular events involved are only poorly understood. *Moringa oleifera* is used for the treatment of diabetes. However, its role on mitochondrial functionality is not yet established. This study was aimed to evaluate the effect of *M. oleifera* extract on supercomplexes formation, ATPase activity, ROS production, GSH levels, lipid peroxidation and protein carbonylation. The levels of lipid peroxidation and protein carbonylation were increased in diabetic group. Whereas, the levels were decreased in *M. oleifera* treated diabetic rats. Analysis In-gel activity showed an increase in all complexes activities in diabetic group, but spectrophotometric determinations of complex II and IV activities were unaffected in this treatment. However, we found an oxygen consumption abolition through complex I-III-IV pathway in diabetic group treated with Moringa. While respiration with succinate feeding into complex II-III-IV was increased in diabetic group. We have shown for the first time that *M. oleifera* extract modulates mitochondrial respiratory activity, an effect that may account for some of the protective properties of phytochemicals. These effects may be of physiological significance since it seems that some phytochemicals are concentrated into mitochondria. The results also support a pharmacological use of *M. oleifera* extract in drug to reduce mitochondrial damage *in vivo*.

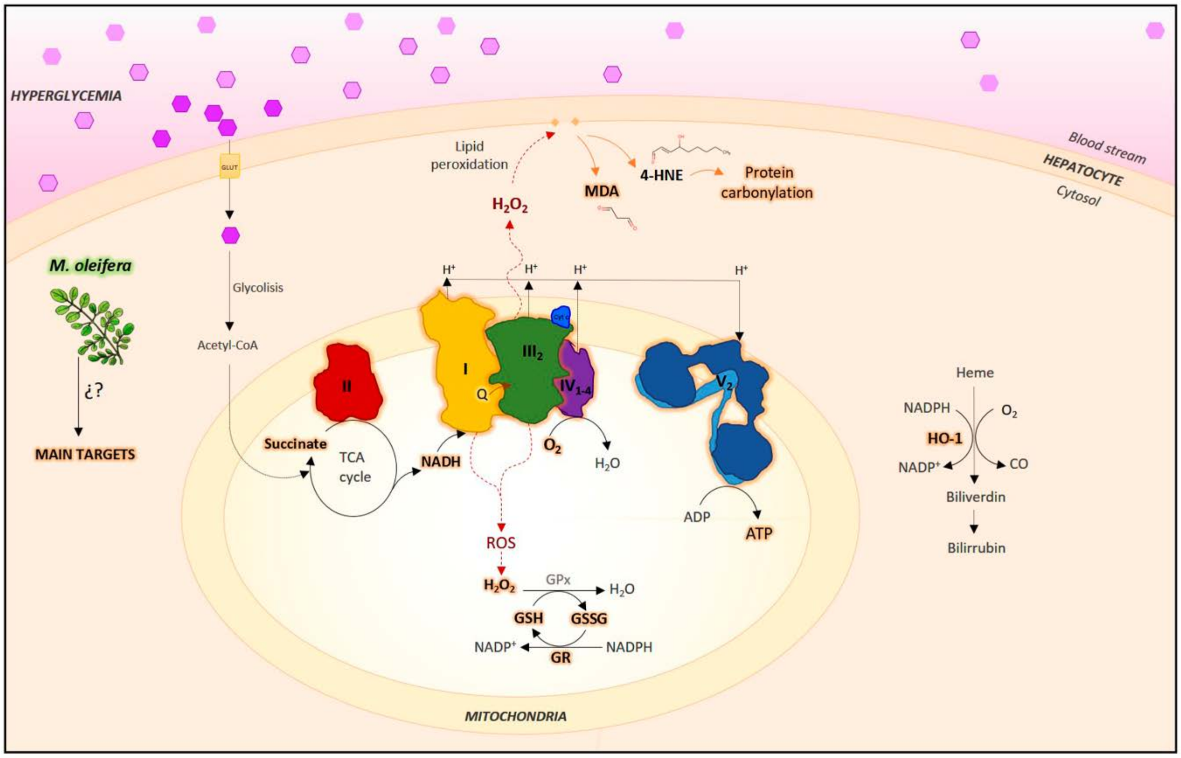

Supplement: Supplementary Materials — Figure S1: Possible mitochondrial processes that are modulated by Moringa oleifera extract. It is now well established that the individual mitochondrial respiratory complexes can be organized into supercomplexes, but the composition and abundance of these may vary among organisms and tissues depending on the metabolic and physiological conditions. Alteration of mitochondrial electron transport chain is a recognized hallmark of the diabetic-associated decline in liver bioenergetics; however, the molecular events involved are only poorly understood. Moringa oleifera is used for the treatment of diabetes. However, its role on mitochondrial functionality is not yet established. This study was aimed to evaluate the effect of M. oleifera extract on supercomplex formation, ATPase activity, ROS production, GSH levels, lipid peroxidation, and protein carbonylation. The levels of lipid peroxidation and protein carbonylation were increased in the diabetic group. However, the levels were decreased in M. oleifera-treated diabetic rats. Analysis of in-gel activity showed an increase in all complexes activities in the diabetic group, but spectrophotometric determinations of complex II and IV activities were unaffected in this treatment. However, we found an oxygen consumption abolition through complex I-III-IV pathway in the diabetic group treated with Moringa. Respiration with succinate feeding into complex II-III-IV was increased in the diabetic group. We have shown for the first time that M. oleifera extract modulates mitochondrial respiratory activity, an effect that may account for some of the protective properties of phytochemicals. These effects may be of physiological significance since it seems that some phytochemicals are concentrated into mitochondria. The results also support a pharmacological use of M. oleifera extract in drug to reduce mitochondrial damage in vivo. [file 5681081.f1.pdf]
